# Middle cerebral artery velocity dynamic response profile during exercise is attenuated following multiple ischemic strokes: a case report

**DOI:** 10.14814/phy2.14268

**Published:** 2019-11-05

**Authors:** Carolyn S. Kaufman, Stephen X. Bai, Jaimie L. Ward, Sarah M. Eickmeyer, Sandra A. Billinger

**Affiliations:** ^1^ Department of Molecular and Integrative Physiology University of Kansas Medical Center Kansas City Kansas; ^2^ Department of Physical Therapy and Rehabilitation Science University of Kansas Medical Center Kansas City Kansas; ^3^ Department of Physical Medicine and Rehabilitation University of Kansas Medical Center Kansas City Kansas; ^4^ Department of Neurology University of Kansas Medical Center Kansas City Kansas

**Keywords:** Cerebrovascular, exercise, stroke, ultrasound

## Abstract

Blood flow regulation is impaired in people with stroke. However, the time course of change in middle cerebral artery velocity (MCAv) following repeated stroke at rest and during exercise remains unknown. In this case study, we provide novel characterization of the dynamic kinetic MCAv response profile to moderate‐intensity exercise before and after repeated ischemic MCA stroke. The initial stroke occurred in the left MCA. At 3 months poststroke, left MCAv amplitude (Amp) was ~50% lower than the right. At the 6‐month follow‐up visit, MCAv Amp declined in both MCA with the left MCAv Amp ~50% lower than the right MCAv Amp. Following a second right MCA stroke, we report further decline in Amp for the left MCA. At the 3‐ and 6‐month visit following the second stroke, the left MCAv Amp declined further (~10%). The right MCAv Amp dramatically decreased by 81.3% when compared to the initial study visit. The MCAv kinetic analysis revealed a marked impairment in the cerebrovascular response to exercise following stroke. We discuss potential pathophysiological mechanisms contributing to poststroke cerebrovascular dysfunction and the need to test therapeutic interventions (such as exercise) that might attenuate cerebrovascular decline in people following stroke.

## Introduction

Cerebral blood flow (CBF) declines as humans age even in the absence of disease (Ainslie et al. 2008). However, age‐related cerebrovascular decline can be attenuated by maintaining aerobic fitness and individuals with the highest levels of aerobic fitness present with the least cerebrovascular decline (Ainslie et al. 2008). Maintaining optimal brain health across the lifespan is essential to reduce the risk of stroke, dementia, and Alzheimer's disease (Gorelick et al. 2017). Poor brain health has been associated with modifiable cardiovascular risk factors such as hypertension, dyslipidemia, and physical inactivity (Gorelick et al. 2011). Further, stroke and associated cardiac risk factors negatively affect vascular health and ultimately influence dementia progression (Gorelick et al. 2011, 2017). Considering the current life expectancy and focus on optimal brain aging (Gorelick et al. 2017), identifying effective tools to assess cerebrovascular health has become increasingly critical.

We published a novel method for measuring cerebrovascular response to a change in demand from rest to moderate‐intensity exercise in the middle cerebral artery (MCA) (Billinger et al. 2017). The resolution of the MCA velocity (MCAv) kinetics provided unique information for age‐ and sex‐differences and revealed that the kinetics profile was blunted in older adults when compared to their younger counterparts (Ward et al. 2018). In addition, older women demonstrated a delayed response following exercise onset. We recently published individuals 3 months after MCA stroke have a significantly lower MCAv during moderate‐intensity exercise than sedentary, age‐, and sex‐matched adults (Kempf et al. 2019). During recruitment for the larger study (Kempf et al. 2019), one of our participants experienced two ischemic strokes. Therefore, the purpose of this case study is to describe the effect of MCA stroke on the cerebrovascular response to an acute bout of moderate‐intensity exercise and to characterize the response over time in this individual.

## Case Report

This individual experienced two ischemic strokes approximately 1 year apart with the first stroke in the left MCA followed by the right MCA. The first study visit occurred at 3 months after the left MCA stroke and the follow‐up visit at 6 months poststroke. The experimental protocol consisted of measuring MCAv, heart rate (HR), mean arterial pressure (MAP), and end‐tidal carbon dioxide (P_ET_CO_2_). This timeline was repeated after the right MCA stroke.

**Figure 1:**
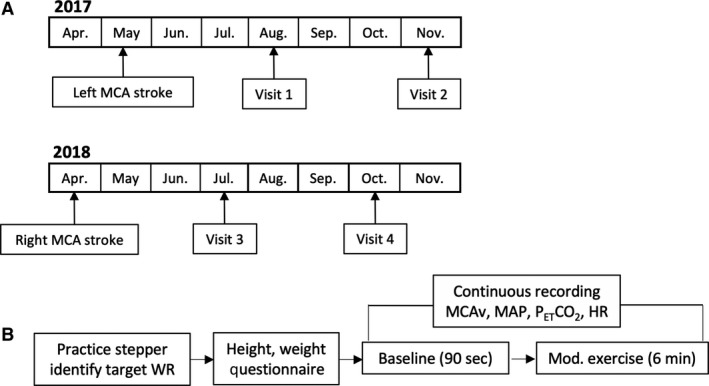
(A) Timeline of stroke and visits to the laboratory. (B) Experimental protocol at each study visit. WR, Work Rate; MCAv, Middle Cerebral Artery velocity; MAP, Mean Arterial Pressure; P_ET_CO_2_, End‐Tidal Carbon Dioxide; HR, Heart Rate.

See Figure 1 for a timeline of stroke events, study visits to our laboratory, and experimental protocol.

### Initial stroke (Left MCA)

This 66‐year‐old male was admitted for stroke in May 2017. The admission exam showed right‐sided weakness, aphasia, and a National Institutes of Health Stroke Scale (NIHSS) score of 10. Computed tomography angiography (CTA) found MCA stenosis of the left M2 segment without large vessel occlusion. Magnetic resonance imaging (MRI) showed a left M2 distribution subacute infarct. The initial stroke was 71.04 cm^3^ (Kothari et al. 1996). Four days poststroke, the patient was discharged to inpatient rehabilitation facility with an NIHSS score of 2.

This patient was recruited during the acute hospital stay and expressed interest in our study. This patient was contacted by phone to schedule Visit 1 at 3 months poststroke and Visit 2 at 6 months poststroke. Prior to each study visit, the participant was asked to abstain from food within 2 h, caffeine for at least 6 h, and vigorous exercise for 12 h. The University of Kansas Medical Center Human Subjects Committee approved all experimental procedures in compliance with the Declaration of Helsinki. Written informed consent was obtained prior to study participation. After consent, we obtained information from the electronic medical record related to the stroke.

### Second stroke (Right MCA)

The stroke admission exam (April 2018) was notable for an NIHSS score of 16. Subsequent CTA showed high‐grade stenosis at the right M1 MCA bifurcation. MRI showed acute right MCA stroke. The second stroke measured 50.11 cm^3^ (Kothari et al. 1996). At 3 days poststroke, the patient was discharged to inpatient rehabilitation and had an NIHSS score of 5.

### Experimental protocol

The participant was familiarized with the equipment and then we determined the work rate (WR) (Kempf et al. 2019) needed to reach moderate‐intensity exercise. Moderate‐intensity exercise was defined as 45–55% of HR reserve, calculated using the Karvonen formula and age predicted HR maximum: target HR = [((220−age)−resting HR) × %intensity) + resting HR]. We administered the questionnaire for physical activity (Jurca et al. 2005). The participant was then seated on the exercise device and the equipment setup commenced.

The sonographer blinded to the side of stroke applied the 2‐MHz transcranial Doppler ultrasound probes bilaterally (Multigon Industries, Yonkers, NY). A five‐lead ECG continuously monitored HR. Beat‐to‐beat blood pressure (BP) was acquired from the left middle finger (Finometer PRO; Finapres Medical Systems, Amsterdam, The Netherlands). A nasal cannula measured P_ET_CO_2_ (in mmHg) (BCI Capnocheck Sleep 9004 Smiths Medical, Dublin, OH).

We acquired 90 sec of baseline (BL) data during seated rest then the participant began the exercise bout on the recumbent stepper (NuStep T5XR, NuStep Inc, Ann Arbor, MI) and exercised at target WR for 6 min. After rest to ensure HR and MCAv returned to BL, the exercise bout was repeated to improve the signal‐to‐noise ratio. Data acquisition occurred through an analog‐to‐digital unit (NI‐USB‐6212, National Instruments) and custom‐written software (MATLAB, v2014a, The Mathworks Inc. Natick, MA). Sampling rate was 500 Hz and interpolated to 2.0 Hz. Three‐second averages were calculated and then smoothed using a 9‐sec sliding window average (Ward et al. 2018; Kempf et al. 2019). To increase the signal‐to‐noise ratio, the two interpolations were averaged over the entire test period and the average response was modeled:

$$\text{MCAv}\left(t\right)=\text{BL}*\left(t\leq \text{TD}\right)+\left(\text{BL}+\text{Amp}\left(1-e^{-(t-\text{TD})/\tau }\right)*(t>\text{TD)}\right)$$

where MCAv(*t*) is the MCAv at any point in time (*t*), BL is baseline (before exercise onset), Amp is the peak amplitude of the response, TD is the time delay preceding the increase in MCAv, and *τ* is the time constant (i.e., time to reach 63% of steady state).

The MCAv data for Visit 1 were published as part of a larger data set (Kempf et al. 2019). In this case study, we include data for all four visits. Participant MCAv kinetic response, MAP, HR, and P_ET_CO_2_ data for each visit are found in Table 1.

**Table 1:**
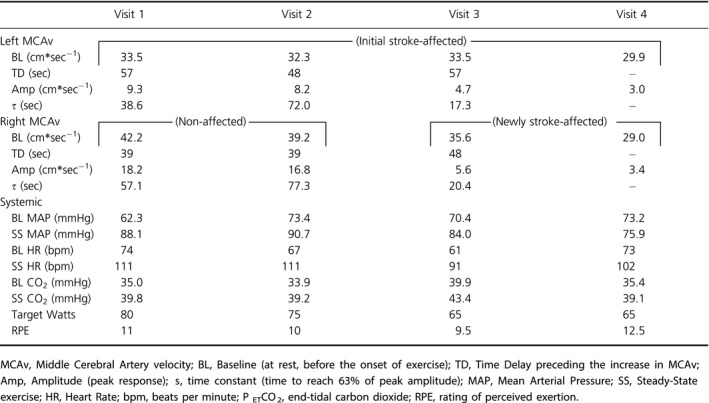
Data from each experimental visit from rest to moderate exercise after left MCA stroke (Visits 1 and 2) and subsequent right MCA stroke (Visits 3 and 4).

### Visit 1 (3 months after left MCA stroke)

BL MCAv and Amp during exercise were lower in the left MCA than the right MCA. Left BL MCAv was 8.7 cm*sec^−1^ lower, which was 20.6% less than the right BL MCAv. In response to moderate‐intensity exercise, the Amp for the left MCA was 48.9% lower than the right MCA. For the left MCAv, TD was 18 sec slower while *τ* responded faster than the right MCAv. Using a self‐report physical activity questionnaire (Jurca et al. 2005), the participant engaged in aerobic activities for 20–60 min per week.

### Visit 2 (6 months after left MCA stroke)

We observed that the left BL MCAv had a 3.6% decrease and the Amp was 11.8% lower than Visit 1. The right BL MCAv also decreased and was 7.1% lower than Visit 1 while Amp was reduced by 7.7%. We observed a slower MCAv kinetics at Visit 2 compared to Visit 1. This was primarily driven by increased time constant, *τ*. The participant reported engaging in physical activity with low levels of exertion for at least 10 min.

### Visit 3 (3 months after right MCA stroke)

Following the right MCA stroke, differences between the left and right BL MCAv were not as pronounced. A 2.1 cm*sec^−1^ (5.9%) difference was observed between left and right BL MCAv and the left MCA Amp was only 0.9 cm*sec^−1^ (16.1%) lower than the right MCA Amp. TD slowed for the right MCAv while *τ* occurred much faster than Visit 2.

### Visit 4 (6 months after right MCA stroke)

**Figure 2:**
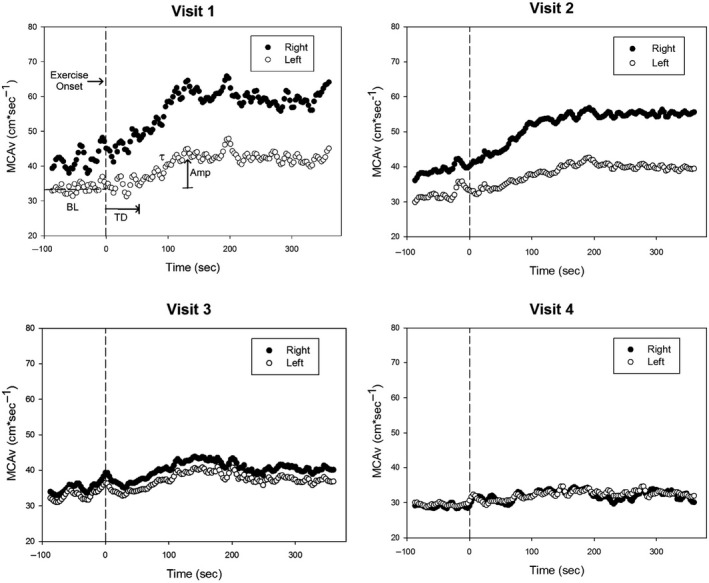
Bilateral Middle Cerebral Artery velocity (MCAv) at rest and during moderate‐intensity exercise. Time point zero indicates the onset of exercise. Key parameters are indicated: BL, Baseline; TD, Time Delay; *τ*, Time Constant which denotes time‐to‐63% of primary response (i.e., Amp, Amplitude of primary response). Please see text for further details.

We observed negligible difference between left and right BL MCAv (0.9 cm*sec^−1^) and Amp (0.4 cm*sec^−1^). The overall decline in left BL MCAv from Visit 1 to Visit 4 was 3.6 cm*sec^−1^ (−10.7%) while the right BL MCAv decreased by 13.2 cm*sec^−1^ (−31.3%). We report a 6.3 cm*sec^−1^ (−67.7%) reduction in Amp for the left MCA while the right MCA Amp was 14.8 cm*sec^−1^ (−81.3%) lower than Visit 1. TD and *τ* were indeterminate. Figure 2 demonstrates the changes in BL and Amp over time. At Visits 3 and 4, the participant reported being inactive other than usual daily activities.

## Discussion

This case study provides novel insight into cerebrovascular changes following MCA stroke. We are the first to highlight the pattern of change in MCAv kinetic response to exercise at 3‐ and 6‐months poststroke. Given the current emphasis on cerebrovascular health and brain aging, these data provide evidence to support interventions targeted at improving vascular contributions to brain health.

### Initial stroke (Left MCA)

We observed a lower BL MCAv in the left (stroke‐affected) MCA than the right (non‐affected) MCA. Left MCAv Amp was ~50% lower and TD was 18.0 sec slower than the right, suggesting cerebrovascular dysfunction at 3 months poststroke. We observed a decline over time in both the stroke‐affected and non‐affected BL and Amp values. By Visit 2, the MCAv kinetic response had slowed, which was largely driven by *τ*. We observed that *τ* slowed (i.e., longer time to reach 63% of steady state) by 33.4 sec in left MCAv and 20.2 sec in the right MCAv. This slower response may be indicative of a delayed vasomotor control response after stroke. The Amp in the stroke‐affected MCA for Visit 1 and Visit 2 was 9.3 cm*sec^−1^ and 8.2 cm*sec^−1^, respectively, while the non‐affected Amp was 18.2 cm*sec^−1^ and 16.8 cm*sec^−1^. These values were higher than those reported for participants' poststroke and sedentary controls in our previous work (Kempf et al. 2019).

### Second stroke (Right MCA)

We observed a reduction in right BL MCAv (−15.6%) and Amp (−69.2%) at Visit 3 compared to the initial Visit 1. While the left BL MCAv did not change (0%), the left Amp decreased 50% from Visit 1. The TD slowed at Visit 3 while *τ* was dramatically shorter (~50 sec) from Visit 2. The reduction in MCAv Amp may be a primary factor for this faster *τ* response. In our original published work, individuals with the largest Amp show increased *τ* values. Although it is difficult to disentangle the factors contributing to changes in the MCAv kinetics, this case study offers insight into the potential unique effects of stroke on these parameters.

At Visit 4, BL and Amp decreased further in both MCAs. As observed in Figure 2, both the right and left BL MCAv and kinetic response profile to exercise appear similar in Visits 3 and 4, highlighting a significant change from Visits 1 and 2. Prior work found an average rate of decline in resting MCAv of 0.76 ± 0.04 cm*sec^−1^ per year for healthy male adults (Ainslie et al. 2008). We observed a greater rate of change following stroke. In this participant, the resting (BL) MCAv declined by 3.2 cm*sec^−1^ (10.7%) in the left (initial stroke‐affected MCA) while the right (second stroke) BL MCAv declined by 13.2 cm*sec^−1^ (31.3%) in just 17 months.

We propose some potential mechanisms that may influence our findings, including up‐ or downstream stenosis and impaired vasodilation in arterioles due to neurovascular uncoupling. First, the participant's initial stroke occurred from left M2 stenosis while the right (second) stroke occurred from MCA bifurcation stenosis. This could potentially contribute to increased downstream vascular resistance and partially explain the lower values at rest and during exercise. Second, acute exercise causes neural activation in areas such as the primary sensorimotor cortex and results in increased blood flow to these regions (Orgogozo and Larsen, 1979; Ogoh and Ainslie, 2009), which is mediated by neurovascular coupling (Venkat et al. 2016). Ischemic stroke damages the neurovascular unit resulting in neurovascular uncoupling (Salinet et al. 2018), a situation in which vasodilatory mediators produced by active neurons fail to result in arteriolar vasodilation (Cai et al. 2017). Thus, the reduced Amp and increased TD observed could partially result from neurovascular unit damage (directly related to the stroke), which fails to couple blood flow to local neuronal activity. These data are the first to characterize longitudinal changes in BL MCAv and Amp following stroke, and it has yet to be determined whether the observed changes are directly due to the stroke or more likely, a combination of factors following stroke. For example, the participant's physical activity levels decreased over time. While it is unknown what effect this decrease in physical activity may have on BL MCAv and Amp following stroke, future research could investigate whether increasing physical activity improves the MCAv kinetic profile.

BL and steady‐state MAP varied at each study visit. We acknowledge that our BL MCAv and the kinetic response may have been related to changes in pressure. However, these findings only partially explain our results. If BP was the primary driver for MCAv then both right and left MCAv would be similarly affected. Further, MAP during exercise decreased ~14% from Visit 1 to 4 while the MCAv Amp in both arteries demonstrated >60% decrease. Another potential mechanism known to influence CBF is partial pressure of carbon dioxide (PaCO_2_) in the arterial blood. As discussed in our prior work, we acknowledge that P_ET_CO_2_ is not identical to PaCO_2_ (Billinger et al. 2017), but we can expect an approximate 3–5 mmHg increase in P_ET_CO_2_ from rest to moderate‐intensity exercise. We observed a consistent increase in P_ET_CO_2_ from rest to exercise at all study visits. Exercise HR was identical (111 bpm) at Visits 1 and 2. However, the WR for the participant to reach the target HR zone was lower for all visits after Visit 1. This could be due to several factors not assessed in this case study such as overall deconditioning or a change in neuromuscular performance during exercise especially following the second stroke.

In summary, this case study provides valuable insight and highlights the importance for characterizing the MCAv kinetic response following stroke and exploring therapeutic interventions to improve cerebrovascular health in this patient population.

## Conflict of Interest

The contents are solely the responsibility of the authors and do not necessarily represent the official views of the NIH, NICHD, or NCATS. Dr. Billinger reports a patent pending (18KU028M‐02)**.** No other authors have conflict of interest to report.
